# Identification of Water-Soluble Polymers through Machine Learning of Fluorescence Signals from Multiple Peptide Sensors

**DOI:** 10.1021/acsabm.3c00736

**Published:** 2023-10-27

**Authors:** Shion Hasegawa, Toshiki Sawada, Takeshi Serizawa

**Affiliations:** Department of Chemical Science and Engineering, School of Materials and Chemical Technology, Tokyo Institute of Technology, 2-12-1-H121 Ookayama, Meguro-ku, Tokyo 152-8550, Japan

**Keywords:** water-soluble polymer, peptide sensor, fluorescence signal, machine learning, identification

## Abstract

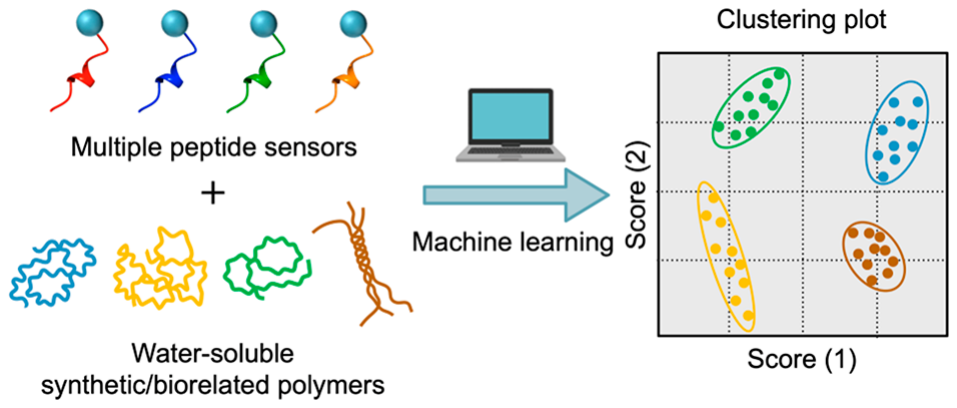

Recently, there has been growing concern about the discharge of water-soluble polymers (especially synthetic polymers) into the environment. Therefore, the identification of water-soluble polymers in water samples is becoming increasingly crucial. In this study, a chemical tongue system to simply and precisely identify water-soluble polymers using multiple fluorescently responsive peptide sensors was demonstrated. Fluorescence spectra obtained from the mixture of each peptide sensor and water-soluble polymer were changed depending on the combination of the polymer species and peptide sensors. Water-soluble polymers were successfully identified through the supervised or unsupervised machine learning of multidimensional fluorescence signals from the peptide sensors.

Water-soluble polymers are widely used in various industries, including water treatment, petroleum refining, paper production, and food processing.^1,2^ Recently, there has been growing concern regarding the discharge of water-soluble polymers (particularly synthetic polymers) into the environment, as these polymers are potentially hazardous to soil and aquatic environments.^3−6^ Therefore, the identification of water-soluble polymers in wastewater or naturally derived water samples is becoming increasingly crucial. However, analytical methods for water-soluble polymers have hardly been proposed. A potential candidate is based on gas chromatography/mass spectrometry of pyrolysis products obtained from solidified samples.^7−9^ However, sample preparation is laborious, and special apparatuses are needed. Therefore, simple and convenient methods are urgently needed to identify water-soluble polymers, particularly in their solution state.

In the mammalian gustatory system, taste cells possess multiple receptor proteins.^10,11^ The characteristic gustatory substances for each taste interact with the receptor proteins to obtain specific signal patterns, which can be recognized for each taste. The system is remarkably performed even though the total numbers of receptor proteins are smaller than those of the gustatory substances. Inspired by biological sensing systems, the development of artificial sensing systems called “chemical tongues”,^12−14^ which emulate the unique mechanism of the mammalian gustatory system, has gained considerable attention; these systems exhibit potential for effectively and precisely identifying chemical species with a limited number of artificial sensors. Thus far, polymers,^15,16^ supramolecules,^17,18^ and nanoparticles^19^ have been used as artificial sensors that interact with sensing targets, including proteins,^20,21^ metal ions,^22,23^ and cells,^13,16^ to generate multiple optical signals. Machine learning of multidimensional signals has been generally used to identify targets.

In our previous study,^24^ water-soluble polymers in their solution state were identified by applying fluorescence signals from a single molecular sensor to machine learning, which consisted of two functional moieties. One is the 12-mer peptide (sequence: His-Gln-Ile-Ala-His-Lys-Ala-Glu-His-Arg-Leu-Arg) moiety that interacts with various water-soluble polymers, and the other is a microenvironment-responsive fluorophore moiety (namely, 1-anilinonaphythalene, AN). The interactions of the peptide moiety with water-soluble polymers in aqueous solutions provided different fluorescence signals for each polymer. However, it was necessary to collect discriminative signals by appropriately changing solution pH values or adding inorganic salts and alcohols to the solutions. To avoid these complicated procedures, we hypothesized that the parallel use of multiple peptide sensors (but not a single peptide sensor), similar to the biological sensing system, could show potential for identifying water-soluble polymers more simply.

In this study, we demonstrate a chemical tongue system to simply and precisely identify water-soluble polymers using fluorescently responsive multiple peptide sensors (Figure 1). As the peptide moiety, we employed not only the previously used sequence^24^ (His-Gln-Ile-Ala-His-Lys-Ala-Glu-His-Arg-Leu-Arg) but also three mutant sequences, in which each His residue was replaced with an Ala residue. We anticipated that replacing His with Ala could modulate hydrogen-bonding and/or hydrophobic interactions of the original peptide with water-soluble polymers because our previous study revealed that the His residues significantly interacted with poly(*N*-isopropylacrylamide) (PNIPAM).^25^ As the microenvironment-responsive fluorophore moiety, the AN unit was conjugated through the thiol group of the Cys residue additionally introduced to the C-terminus of each peptide, similar to our previous study.^24^ The peptide sensors were named Original, H1A, H5A, and H9A, respectively (Figure 1a). Water-soluble polymers were successfully identified through the supervised or unsupervised machine learning of multidimensional fluorescence signals from the peptide sensors (Figure 1b).

**Figure 1:**
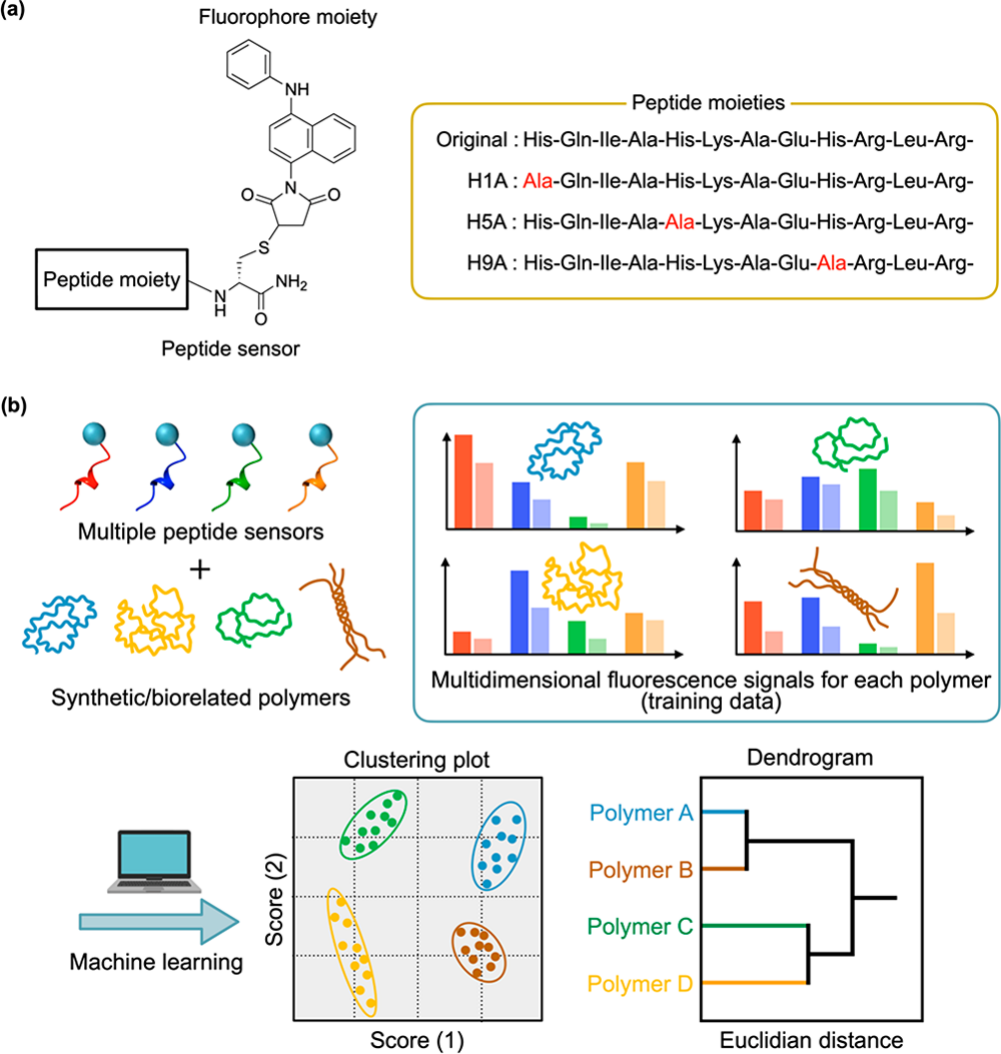
(a) Peptide sensor design for chemical tongues. (b) Schematic illustration of polymer classification and identification through machine learning of fluorescence signals from multiple peptide sensors.

## Fluorescence Spectra of Peptide Sensors in the Presence of Water-Soluble Polymers

**Figure 2:**
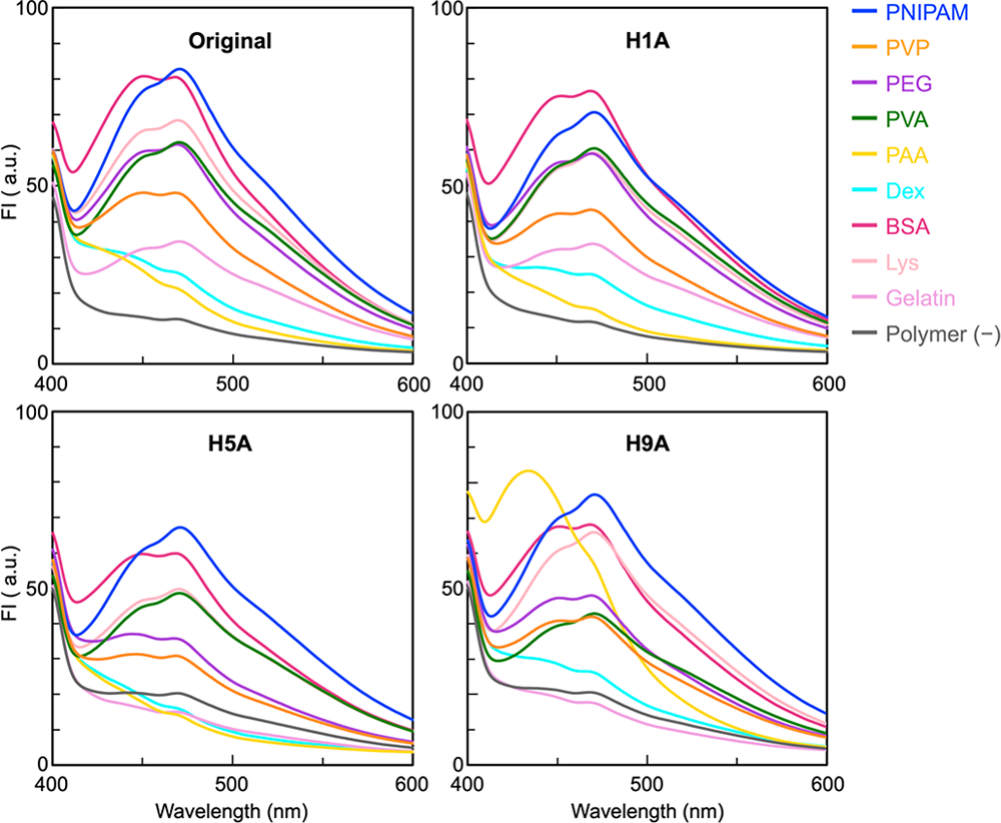
Fluorescence spectra of the peptide sensors in the presence or absence of water-soluble polymers under neutral solution conditions (pH 7.0). The concentrations of the peptide sensor and each polymer were 1 μM and 10 μg mL^–1^, respectively.

Experimental details are shown in the Supporting Information (SI). Synthetic and biorelated water-soluble polymers, such as poly(*N*-isopropylacrylamide) (PNIPAM), poly(vinylpyrrolidone) (PVP), poly(ethylene glycol) (PEG), poly(vinyl alcohol) (PVA), poly(acrylic acid) (PAA), dextran (Dex), bovine serum albumin (BSA), lysozyme (Lys), and gelatin, were used in this study, similar to our previous study.^24^ Some synthetic polymers are widely used in industries and/or daily life. Fluorescence spectra of the mixture solutions containing each polymer (10 mg L^–1^) and each peptide sensor (1 μM) in Britton–Robinson (BR) buffer (pH 7.0) were measured with an excitation wavelength of 350 nm at 25 °C (Figure 2). The spectra from the four peptide sensors in the absence of polymers were slightly different (Figure S1), suggesting that the AN moieties of the sensors are found in different microenvironments. However, circular dichroism (CD) spectra for the four sensors were almost the same (Figure S2), suggesting that the peptide moieties for the four sensors do not exhibit remarkable differences in the secondary structures.

The fluorescence spectra obtained from the peptide sensors drastically changed in the presence of polymers, suggesting that the sensors interacted with the polymers in their aqueous solutions to generate fluorescence signals. In many cases, the fluorescence intensities increased in the presence of polymers. The wavelength of the fluorescence peaks also changed, depending on the polymer species; however, the changes were minor in almost all cases. In addition, some of the spectra show a shoulder peak. Importantly, the spectra obtained for the same polymer were different among the four peptide sensors (Figure S1). These observations clearly indicated that polymer- and sensor-species-dependent fluorescence spectra were successfully obtained.

## Linear Discriminant Analysis (LDA) of the Fluorescence Signals

**Figure 3:**
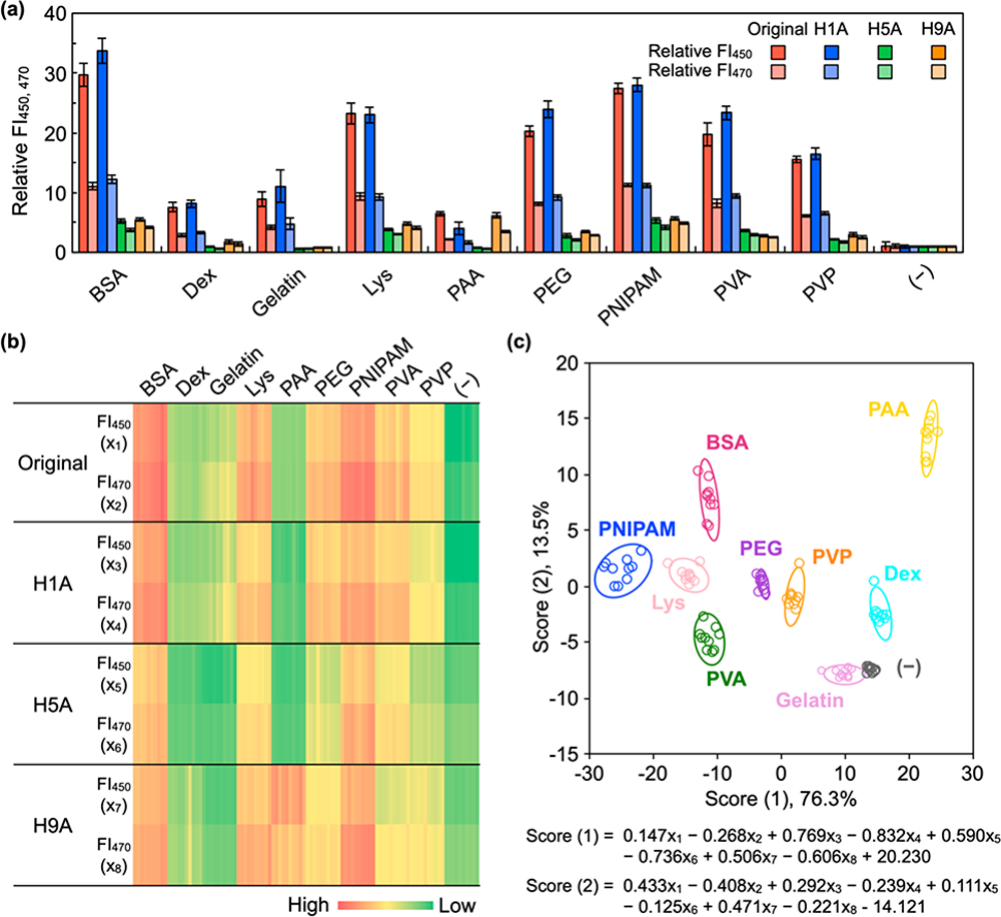
Polymer classification by discrimination of multiple fluorescence signals. (a) Fluorescence intensities relative to those in the absence of polymers at 450 and 470 nm under neutral solution conditions (pH 7.0). The concentrations of the peptide sensor and each polymer were 1 μM and 10 μg mL^–1^, respectively. Each experiment was repeated ten times, and the error bars represent the standard deviation. (b) Heatmap representation of the eight fluorescence signals from the peptide sensors. (c) LDA score plot for all polymers. The training datasets for each polymer are composed of ten plots, and 95% confidence ellipses are shown for each polymer.

To collect discriminative signals from the spectra, the fluorescence intensities relative to those in the absence of water-soluble polymers at two wavelengths (450 and 470 nm) were obtained in the presence and absence of water-soluble polymers using the four peptide sensors; thus, the eight signals (that is, two relative intensities × four peptide sensors) for each polymer were collected (Figure 3a). The wavelengths were selected because the main and shoulder fluorescence peaks were observed around the latter and former wavelengths for many polymers, respectively. The experiments were performed ten times to obtain the ten datasets. To visualize the results, the eight signals for the 10 datasets are shown in the form of a heatmap (Figure 3b), clearly indicating that the four peptide sensors provided different signal patterns for each polymer. To analyze the signal patterns through a machine-learning approach, LDA, which is the most commonly used supervised machine learning for data classification and dimensionality reduction,^26,27^ was applied to the ten datasets as the training datasets for all polymers (namely, each set is composed of the eight-dimensional training data), and then a two-dimensional plot (referred to as the LDA score plot) was obtained (Figure 3c). The first two discriminant scores [namely, score (1), 76.3%; score (2), 13.5%] in the resulting discriminant function accounted for approximately 90% of the total variance, indicating that most of the information in the training datasets is involved in the LDA score plot.

The ten scores for each polymer and only peptide sensors in the plot are enclosed in ellipses with a 95% confidence level to classify the scores into each cluster. The confidence ellipses demonstrated that the ten clusters were well separated, suggesting that there are statistically significant differences in the signal patterns. When the LDA score plots were obtained from different combinations of only three peptide sensors, overlap of some clusters was observed (Figure S3). When H5A was excepted, there was no overlap of the clusters; however, the distances between clusters were closer than those for the original plot obtained from four peptide sensors. These results indicate the superiority of employing multiple peptide sensors (namely, four peptide sensors in the present study). Furthermore, when the LDA score plots for four peptide sensors were obtained at a single wavelength (450 or 470 nm; namely, four-dimensional training data), the classification capability was clearly diminished compared to that of the original plot (Figure S4), emphasizing the superiority of employing two wavelengths. Thus, the eight-dimensional training data from four peptide sensors at two different wavelengths were valuable in identifying water-soluble polymers.

## Hierarchical Cluster Analysis (HCA) of the Fluorescence Signals

**Figure 4:**
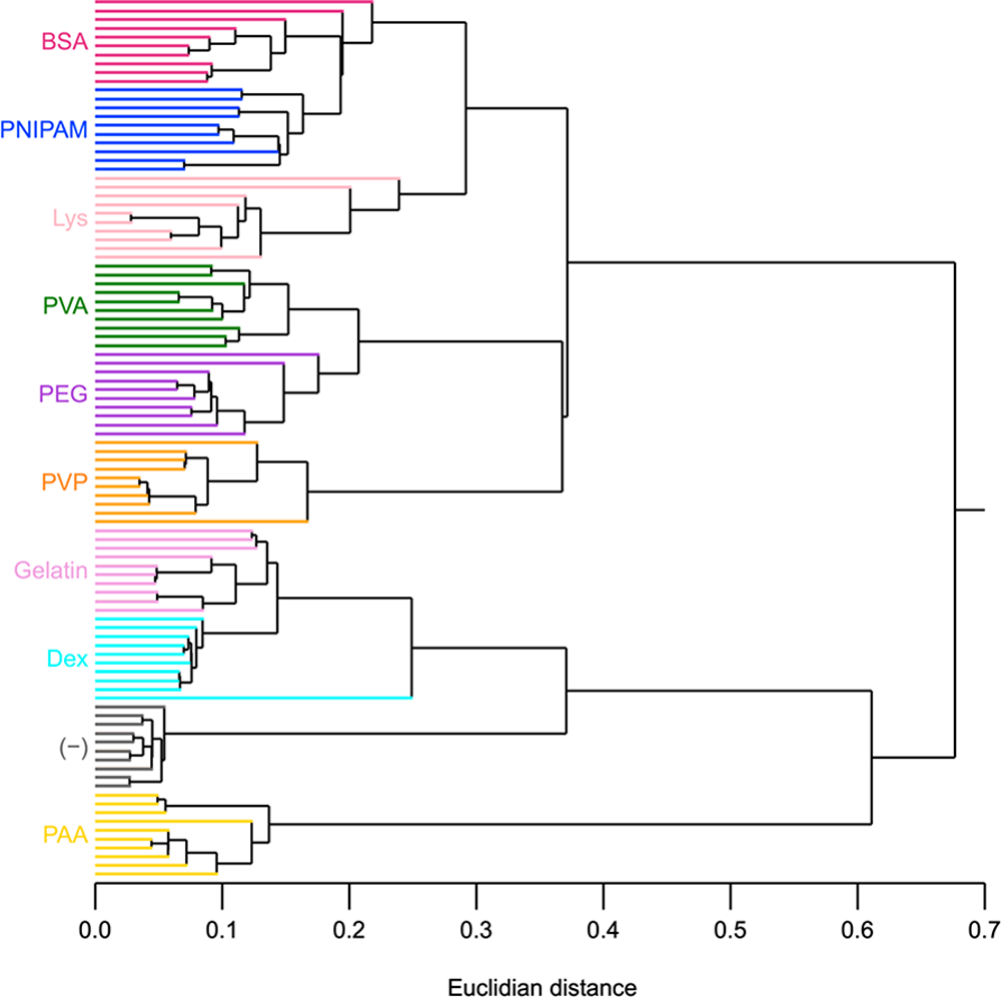
HCA of the ten datasets for each polymer.

To classify the same training datasets through a different machine-learning approach, HCA^26,28^ was applied, which is an algorithmic approach used to identify discrete groups with varying degrees of similarity in a dataset based on unsupervised classification according to their spatial distances (that is, the Euclidean distance; Figure 4). Notably, the smaller distance (that is, the X axis) in the dendrogram indicates more similar classes. Remarkably, the ten datasets for each polymer were in close proximity in almost all cases; the exceptions are two BSA datasets and one Dex dataset, which correspond to only ∼3% of all of the datasets. This result indicated successful clustering of the datasets based on polymer species. In our previous study,^24^ the PNIPAM cluster was significantly far from other clusters when a single peptide sensor (that is, Original) was used, reflecting the specificity of the peptide moiety for PNIPAM. In the present study, the PNIPAM cluster was closer to some polymers, including BSA, indicating that the application of multiple peptide sensors changed the classification mechanism, possibly due to diversification of interactions with polymers. In other words, three newly applied peptide sensors (that is, H1A, H5A, and H9A) appeared to nonspecifically interact with the present polymers in diverse ways.

In the previous section, the similarity of water-soluble polymers was evaluated through HCA of the fluorescence signals (namely, the fluorescence intensities at 450 and 470 nm) from four peptide sensors. In contrast, the similarity of the fluorescence signals can also be evaluated by replacing the training data. To this end, the 10-time-performed data of fluorescence intensities at 450 and 470 nm obtained in the presence and absence of nine polymers (that is, 100-dimensional training data) were used as the training data sets for HCA (Figure S5). The original and H1A peptide sensors were in close proximity, indicating that the closer sensors provided similar signals. Furthermore, the signals of the original and H1A peptides were nested, suggesting that the fluorescence intensities at different wavelengths are valuable for collecting diverse signals.

## Validation of the Generalization Performance of Learning Models

To validate the generalization performance of learning models (namely, discriminant functions) for the identification of water-soluble polymers, two cross-validations were conducted. One is leave-one-out cross-validation (LOOCV),^29^ in which one dataset for a certain polymer was excluded from the ten training datasets to use as the test dataset. After the LDA score plot and ellipses with a 95% confidence level were produced, the test dataset was fitted to the resulting discriminant function, and the obtained plot was classified into an ellipse with the shortest Mahalanobis distance. This process was performed for all datasets (namely, one hundred datasets). Significantly, each test dataset was correctly classified into the corresponding polymer. The other is the hold-out method, in which the ten datasets for each polymer were randomly divided into seven training and three test datasets. The seven scores in the LDA score plot were enclosed in ellipses with a 95% confidence level (Figure S6). Then, the three test datasets for each polymer were fitted to the obtained discriminant function, and the resulting plots for each polymer were classified into an ellipse with the shortest Mahalanobis distance (Figure S7). Significantly, all test data plots (three test data plots with or without each polymer) were correctly classified into the corresponding polymers, clearly demonstrating the successful identification of the polymers.

To further evaluate the generalization performance of the classification models, stratified *k*-fold cross-validations (SKCV) (3- and 5-fold)^30^ were conducted. To this end, one-third and one-fifth datasets for each polymer were used as the test datasets for polymer classification, and all datasets were used as the test datasets. The resulting classification accuracy was 100% for each polymer (Table S1), suggesting that the polymer classification was significantly reliable.

We demonstrated a chemical tongue system capable of identifying water-soluble polymers by applying fluorescence signals from multiple peptide sensors to machine learning. The peptide sensors provided polymer-species-dependent fluorescence spectra. Water-soluble polymers were successfully classified in the two-dimensional clustering plot by applying fluorescence intensities at two wavelengths to supervised machine learning, LDA. The polymers were also classified by applying unsupervised machine learning, HCA, to the same training dataset. Classification reliability and applicability to polymer identification were validated by LOOCV, the hold-out method, and SKCV. For practical applications to water-soluble polymers in the environment, the fluorescence signals based on ratios of appropriate fluorescence intensities possibly obtained from multiple peptide sensors with different fluorophores might be more helpful. Our identification system, which capitalized on the interactions between peptides and water-soluble polymers to obtain multidimensional signals from the polymers for machine learning, serves as a precursor to the development of a universal system for the identification of a wide range of polymeric materials.

## Supporting Information Available

Experimental section, fluorescence spectra of the peptide sensors in the presence or absence of water-soluble polymers, CD spectra of the four peptide sensors, LDA score plots from different combinations of only three peptide sensors, LDA score plots for four peptide sensors at a single wavelength, HCA for the fluorescence signals, LDA score plot for water-soluble polymers, canonical scores for thirty blind samples on the LDA score plot, and classification accuracy of stratified *k*-fold cross-validation (PDF)
