# Supplementary material for: Identification of Water-Soluble Polymers through Machine Learning of Fluorescence Signals from Multiple Peptide Sensors
Source: ACS Appl Bio Mater. 2023 Oct 27;6(11):4598–602. doi: 10.1021/acsabm.3c00736 (PMC10664068; doi:10.1021/acsabm.3c00736)
Supplement: Supplementary file 1 — mt3c00736_si_001.pdf [file mt3c00736_si_001.pdf]

**Supporting Information**

# Identification of Water-Soluble Polymers through Machine Learning of Fluorescence Signals from Multiple Peptide Sensors

*Shion Hasegawa, Toshiki Sawada, and Takeshi Serizawa**

Department of Chemical Science and Engineering, School of Materials and Chemical Technology, Tokyo Institute of Technology, 2-12-1 Ookayama, Megruo-ku, Tokyo 152-8550, Japan

*Email: serizawa@mac.titech.ac.jp

TABLE OF CONTENTS Page


**Experimental Section** **S3**

**Figure S1.** Fluorescence spectra of the peptide sensors in the presence or absence of water-soluble polymers. The concentrations of each peptide sensor and each polymer were 1 μM and 10 mg $L^{-1}$, respectively. **S6**

**Figure S2.** Circular dichroism (CD) spectra of the four peptide sensors. **S7**

**Figure S3.** LDA score plots from different combinations of only three peptide sensors. **S8**

**Figure S4.** LDA score plots for four peptide sensors at a single wavelength. (a) 450 nm, (b) 470 nm. **S9**

**Figure S5.** HCA for the fluorescence signals. **S9**

**Figure S6.** LDA score plot for water-soluble polymers. Seven training datasets were used, and 95% confidence ellipses are shown for each polymer. **S10**

**Figure S7.** Canonical scores for thirty blind samples on the LDA score plot, which were prepared using the seven datasets randomly selected from ten datasets for each polymer. See Figure S6 for the plot used for 95% confidence ellipses. **S10**

**Table S1.** Classification accuracy of stratified k-fold cross-validation (SKCV) (3-fold and 5-fold) in each case. **S11**

**References** **S12**

**Experimental Section**

**Materials.** Poly(vinyl pyrrolidone) (PVP) ($M_w$ 55,000), poly(ethylene glycol) (PEG) ($M_w$ 35,000), poly(vinyl alcohol) (PVA) ($M_w$ 89,000–98,000), dextran (Dex) ($M_w$ 450,000–650,000), poly(acrylic acid) (PAA) ($M_w$ 25,000), lysozyme (Lys) (from chicken egg white), and gelatin (from porcine skin powder, gel strength ~300 g Bloom, Type A) were purchased from Sigma–Aldrich (Tokyo, Japan). Bovine serum albumin (BSA) (protease-free) was purchased from Wako (Tokyo, Japan). The monomer *N*-isopropyl acrylamide (NIPAM) was kindly supplied by KOHJIN Film & Chemicals Co., Ltd. (Tokyo, Japan). (*N*-(1-anilinonaphytyl-4-maleimide) ANM was purchased from Shanghai iChemical Technologies Co., Ltd. (Shanghai, China). NovaSynTGR resin and 9-fluorenylmethyloxycarbonyloxycarbonyl (Fmoc) amino acid derivatives were purchased from Merck (Darmstadt, Germany). Other reagents were purchased from Nacalai Tesque, Inc. (Kyoto, Japan). Ultrapure water with resistivity greater than 18.2 MΩ cm was supplied by a Milli-Q Advantage A-10 unit, Merck (Darmstadt, Germany).

**Peptide Synthesis.** Free *N*-terminal and amidated *C*-terminal peptides were synthesized by standard solid-phase synthesis using an Fmoc-based strategy based on a standard protocol.[1] In brief, the peptide chains were assembled on NovaSynTGR resin (amino group of 0.25 mmol $g^{-1}$) using Fmoc amino acid derivatives. To cleave the peptides from the resin and remove the protecting groups from their side chains, the resins were treated with trifluoroacetic acid (TFA)/1,2-ethanedithiol/thioanisole/*m*-cresol (10/0.75/0.75/0.25, v/v/v/v) for 3 h. The peptides were purified by reverse-phase high-performance liquid chromatography (HPLC; ELITE LaChrom, Hitachi High-Tech Corporation, Tokyo, Japan) using a COSMOSIL 5C18-AR-300 packed column (20 × 250 mm, Nacalai Tesque, Inc., Kyoto, Japan) with a linear gradient from 99.9% $H_2O$/0.1% TFA to 99.9% acetonitrile/0.1% TFA at a flow rate of 6 mL $min^{-1}$. For ANM

conjugation, ANM (5.7 mg, 18 μmol) was mixed with the corresponding peptide with the additional *C*-terminal Cys (14 μmol) dissolved in dimethyl sulfoxide and the mixture was stirred for 2 h at ambient temperature. The ANM-introduced peptides were purified by HPLC.

**PNIPAM Synthesis.** The polymers used in previous studies were used.[2] Atomic transfer radical polymerization of NIPAM was performed following a previous report. In brief, a NIPAM monomer was purified by recrystallization with hexane beforehand. NIPAM (17.3 mmol), $Me_6TREN$ (3.6, 36, or 360 μmol), and MCP (3.6, 36, or 360 μmol) were dissolved in a 1/4 (v/v) mixture of pure water and ethanol (4 mL), and the solution was degassed by nitrogen bubbling for 5 min. CuCl (3.6, 36, or 360 μmol) was then added, and the mixture was stirred for 1 h at ambient temperature. The reaction mixture was diluted by ethyl acetate, and the resultant polymers were purified by silica gel chromatography and reprecipitated with cold diethyl ether and hexane. After the mixture was dried under reduced pressure, PNIPAM was collected as a white solid. The synthesized PNIPAM was characterized by $^1H$ nuclear magnetic resonance spectroscopy (AVANCE III HD500, Bruker Corporation, Yokohama, Japan) in chloroform-$d_6$. The molecular weight of PNIPAM was determined by SEC (HLC-8120 Gel Permeation Chromatography System, Tosoh Corporation, Tokyo, Japan) equipped with TSKgel GMHXL and G2000HXL columns (Tosoh Corporation, Tokyo, Japan) through ultraviolet and refractive index detection using *N,N*-dimethylformamide containing 10 mM LiBr as an eluent at a flow rate of 1.0 mL $min^{-1}$ at 40 °C.

**Fluorescence Measurement.** Polymers were dissolved in 35 μL of BR buffer (pH 7.0), pH-adjusted BR buffer solutions prepared from phosphoric acid (40 mM), acetic acid (40 mM), and boric acid (40 mM). Thirty-five microliters of Peptide-ANM (1 μM in the same solvent) was mixed with the polymer solutions, and the mixture solutions were incubated for 90-110 min at 25 °C. The fluorescence spectra of the mixture solutions in a quartz cell (3 × 3 × 35 mm) at an excitation

wavelength of 350 nm were obtained using a fluorescence spectrophotometer (FP-6500, JASCO Corporation, Tokyo, Japan) at 25 °C.

**Circular Dichroism (CD) Measurements.** The CD spectra of the peptide sensors dissolved in sodium-phosphate-buffer solutions (10 mM phosphate, pH 7.4) at a concentration of 100 μM were recorded on a CD spectrometer (J-725, JASCO) using a UV cell with 0.2 cm of optical path length under a $N_2$ atmosphere at 25 °C using a wavelength range of 190-250 nm with a resolution of 0.5 nm and a scanning speed of 50 nm $min^{-1}$. The CD spectra were co-added for 4 times.

**Polymer Classification and Identification.** LDA and HCA were performed using the SYSTAT 13 program (Systat Software Inc., San Jose, USA). For LDA, the fluorescence signals for each polymer were transferred to the canonical scores setting with the polymer species as the classifying variable. The canonical scores were plotted with 95% confidence ellipses. HCA dendrograms were created based on Euclidean distances using the Ward method, and a dataset was standardized before analysis using the following equation: $z = (x - \mu)/\sigma$, where z is the standardized score, x is the raw score, μ is the population mean, and σ is the population standard deviation. For LOOCV, one dataset for a certain polymer was excluded to use as a test dataset. The test data were classified into an ellipse generated by the remaining training datasets according to their shortest Mahalanobis distances. This process was performed for all datasets (namely, one hundred datasets). For the hold-out method, three datasets for each polymer were randomly selected from ten datasets as the test datasets. The LDA score plot and ellipses with a 95% confidence level were produced using the remaining seven training datasets. The test data were classified into an ellipse with the shortest Mahalanobis distance. For SKCV, one-third (3-fold) and one-fifth (5-fold) datasets for each polymer were used as the test datasets, and all test data were classified in the same way as LOOCV.

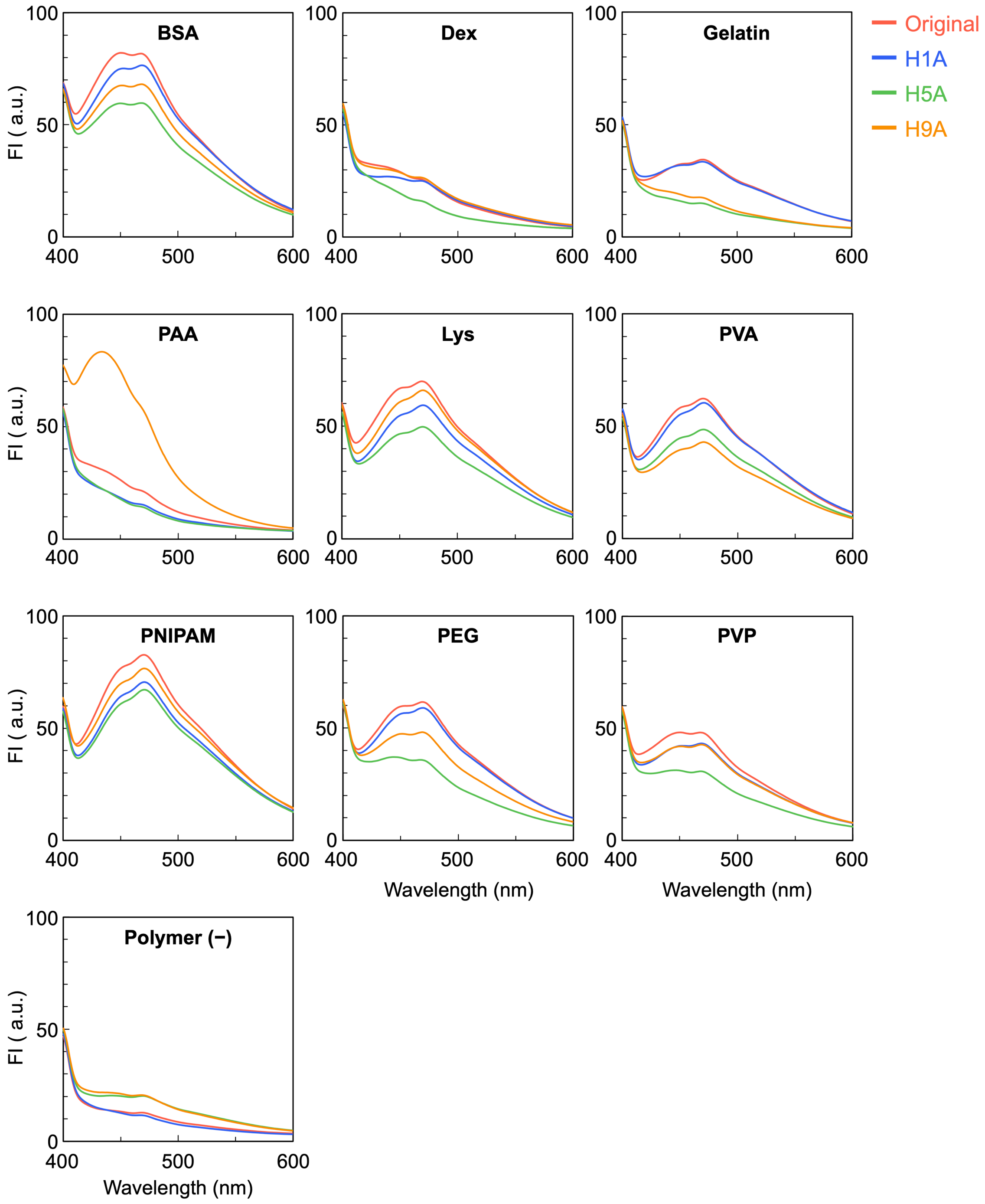


**Figure S1.** Fluorescence spectra of the peptide sensors in the presence or absence of water-soluble polymers. The concentrations of each peptide sensor and each polymer were 1 μM and 10 mg $L^{-1}$, respectively.

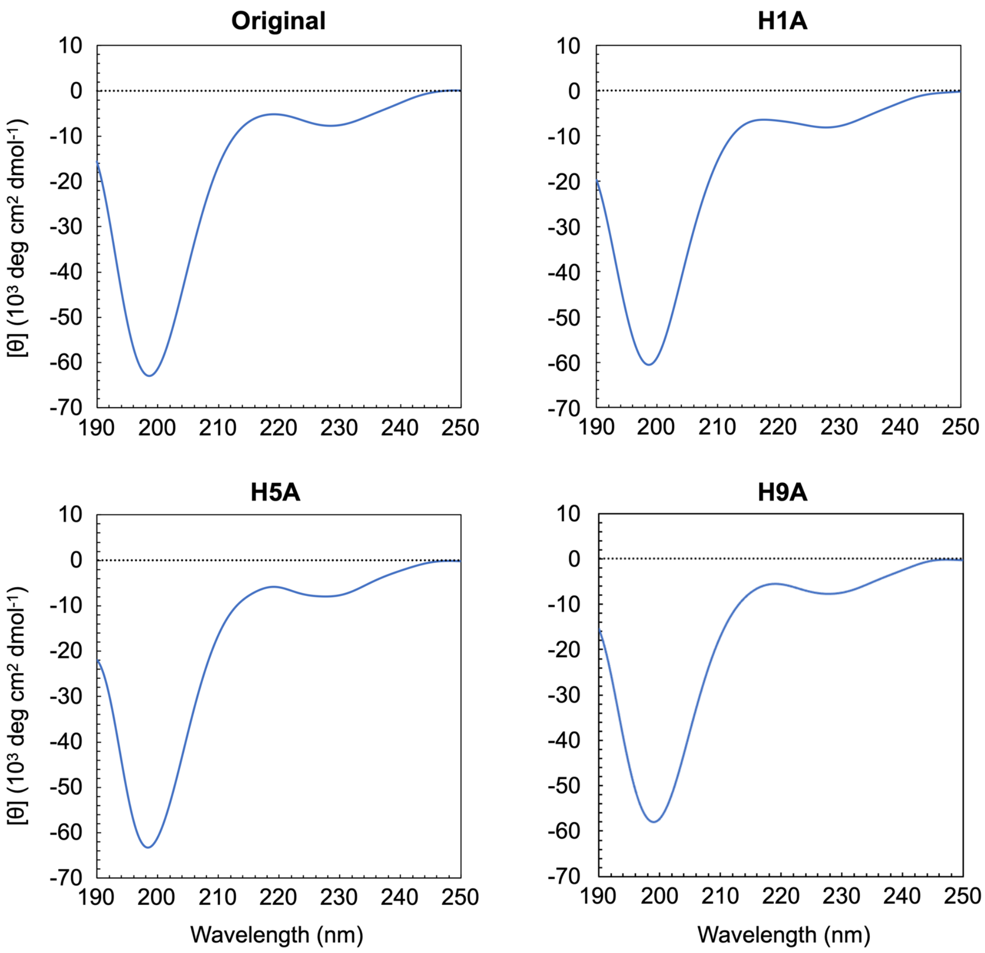


**Figure S2.** Circular dichroism (CD) spectra of the four peptide sensors.

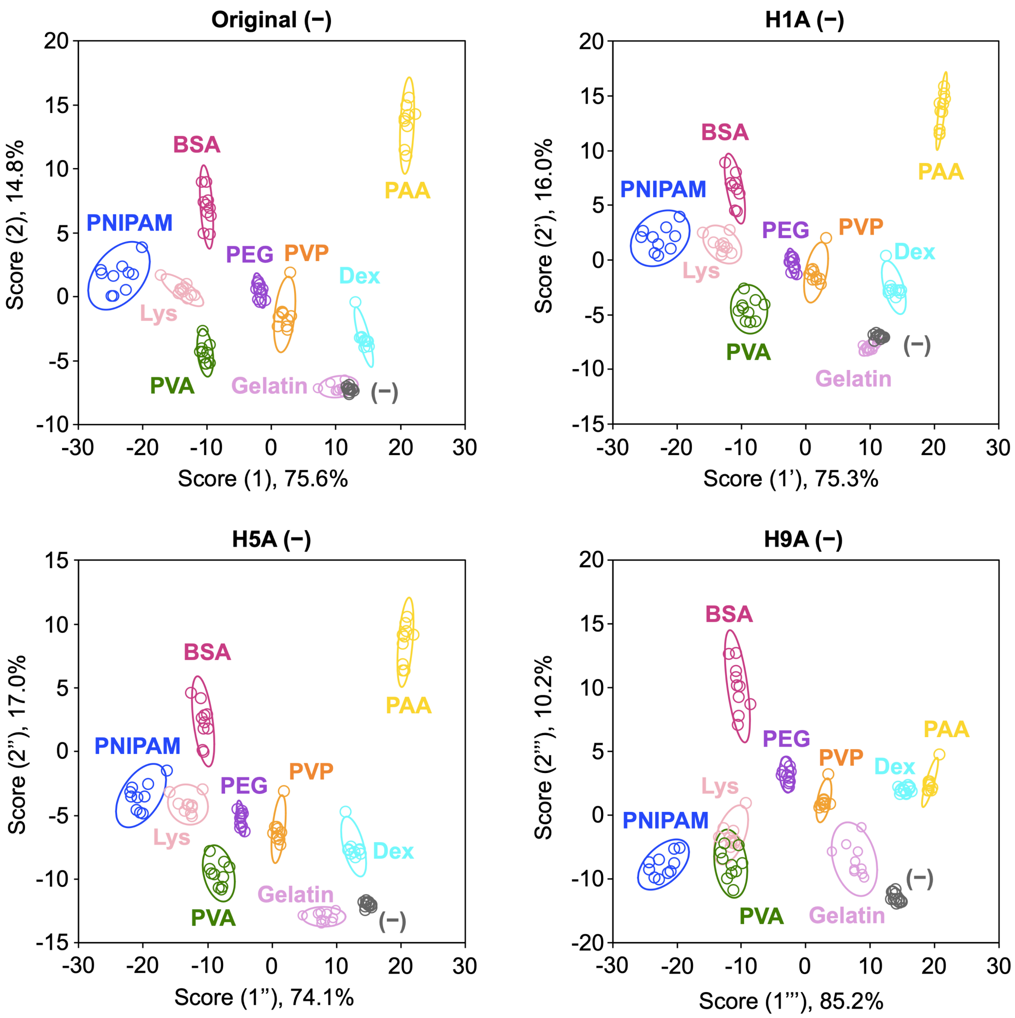


$$\text{Score (1)} = 0.799x_3 - 0.895x_4 + 0.657x_5 - 0.825x_6 + 0.551x_7 - 0.692x_8 + 18.302$$

$$\text{Score (2)} = 0.437x_3 - 0.372x_4 + 0.198x_5 - 0.195x_6 + 0.574x_7 - 0.329x_8 - 13.901$$

$$\text{Score (1')} = 0.318x_1 - 0.453x_2 + 0.488x_5 - 0.679x_6 + 0.607x_7 - 0.713x_8 + 18.656$$

$$\text{Score (2')} = 0.502x_1 - 0.473x_2 + 0.054x_5 - 0.066x_6 + 0.479x_7 - 0.216x_8 - 13.075$$

$$\text{Score (1'')} = 0.174x_1 - 0.324x_2 + 0.735x_5 - 0.830x_6 + 0.604x_7 - 0.744x_8 + 22.395$$

$$\text{Score (2'')} = 0.446x_1 - 0.430x_2 + 0.290x_5 - 0.247x_6 + 0.505x_7 - 0.259x_8 - 3.831$$

$$\text{Score (1''')} = 0.331x_1 - 0.466x_2 + 0.807x_3 - 0.904x_4 + 0.683x_5 - 0.862x_6 + 19.447$$

$$\text{Score (2''')} = 0.616x_1 - 0.493x_2 + 0.865x_3 - 0.708x_4 + 0.748x_5 - 0.748x_6 - 11.031$$

**Figure S3.** LDA score plots from different combinations of only three peptide sensors.

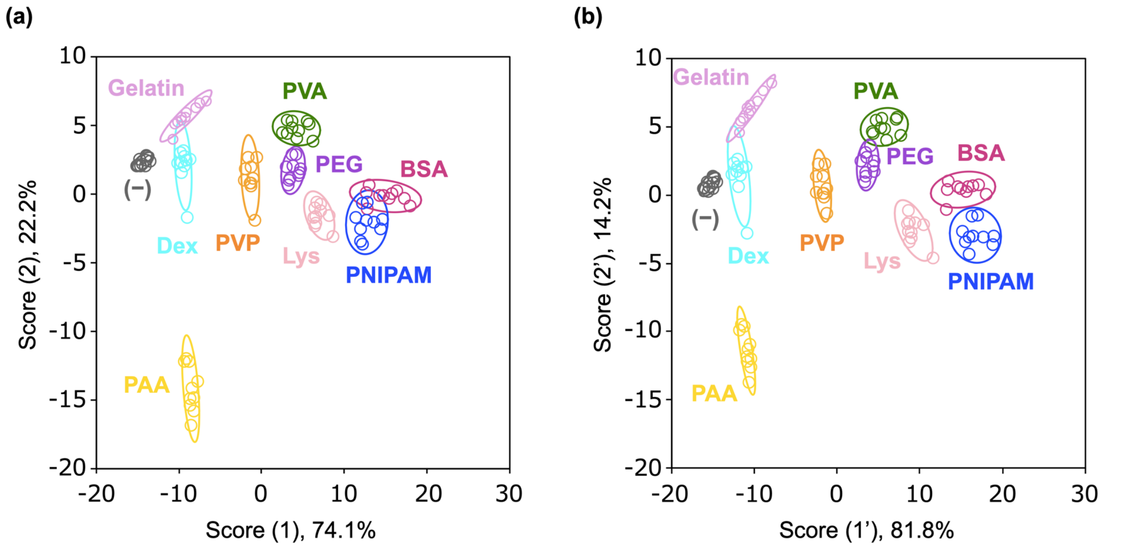


Score (1) = $0.153x_1 + 0.158x_3 + 0.156x_5 + 0.061x_7 - 22.822$

Score (2) = $0.072x_1 + 0.080x_3 + 0.073x_5 - 0.331x_7 + 5.980$

Score (1') = $0.141x_2 + 0.142x_4 + 0.173x_6 + 0.103x_8 - 24.419$

Score (2') = $0.098x_2 + 0.120x_4 + 0.051x_6 - 0.357\,x_8 + 4.437$

**Figure S4.** LDA score plots for four peptide sensors at a single wavelength. (a) 450 nm, (b) 470 nm.

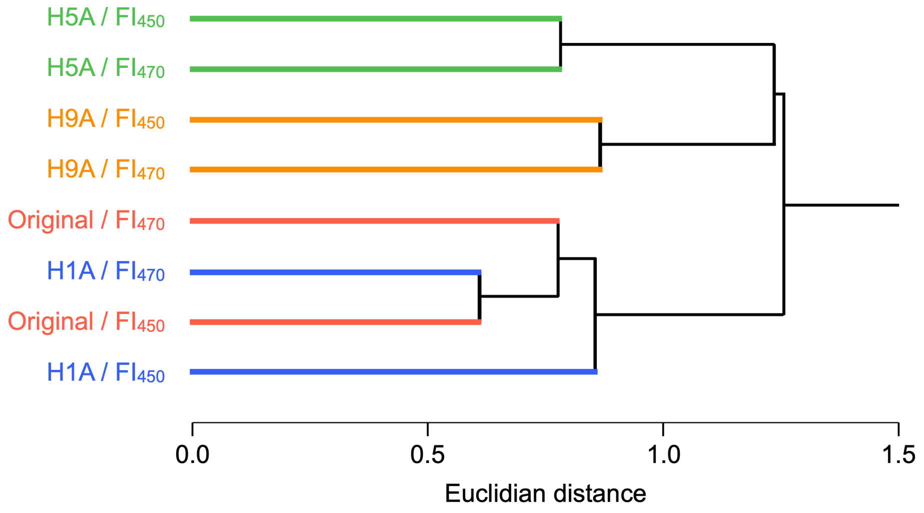


**Figure S5.** HCA for the fluorescence signals.

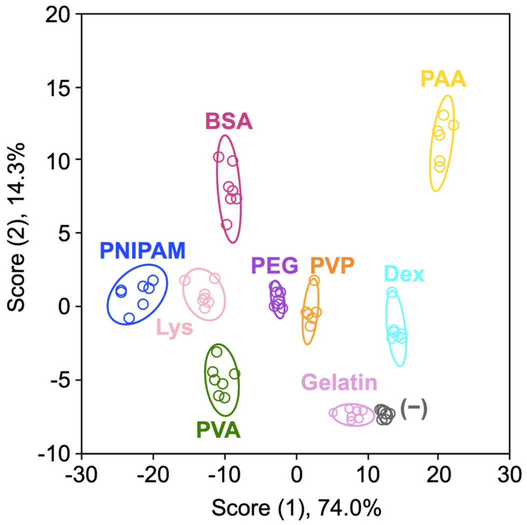


Score (1) = $0.155x_1 - 0.289x_2 + 0.823x_3 - 0.846x_4 + 0.582x_5 - 0.704x_6 + 0.392x_7 - 0.471x_8 + 17.363$

Score (2) = $0.419x_1 - 0.387x_2 + 0.417x_3 - 0.351x_4 + 0.252x_5 - 0.278x_6 + 0.337x_7 - 0.103x_8 - 13.659$

**Figure S6.** LDA score plot for water-soluble polymers. Seven training datasets were used, and 95% confidence ellipses are shown for each polymer.

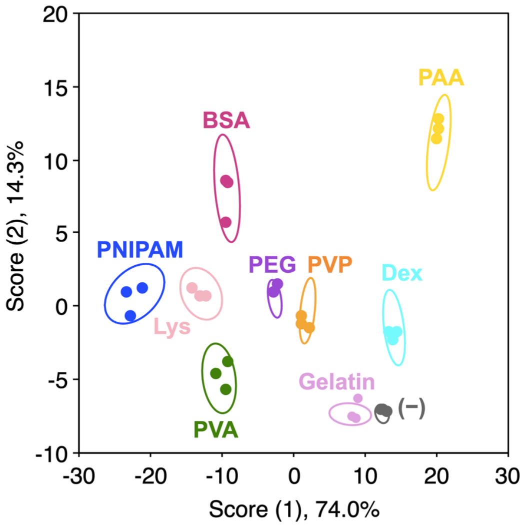


| Polymer | Classification |
|---|---|
| PNIPAM | 3 / 3 |
| Lys | 3 / 3 |
| PVP | 3 / 3 |
| PEG | 3 / 3 |
| PVA | 3 / 3 |
| Dex | 3 / 3 |
| PAA | 3 / 3 |
| BSA | 3 / 3 |
| Gelatin | 3 / 3 |
| (−) | 3 / 3 |

Score (1) = $0.155x_1 - 0.289x_2 + 0.823x_3 - 0.846x_4 + 0.582x_5 - 0.704x_6 + 0.392x_7 - 0.471x_8 + 17.363$

Score (2) = $0.419x_1 - 0.387x_2 + 0.417x_3 - 0.351x_4 + 0.252x_5 - 0.278x_6 + 0.337x_7 - 0.103x_8 - 13.659$

**Figure S7.** Canonical scores for thirty blind samples on the LDA score plot, which were prepared using the seven datasets randomly selected from ten datasets for each polymer. See Figure S6 for the plot used for 95% confidence ellipses.

**Table S1.** Classification accuracy of stratified k-fold cross-validation (SKCV) (3-fold and 5-fold) in each case.

| Polymer | 3-fold / % | 5-fold / % |
|---|---|---|
| PNIPAM | 100 | 100 |
| Lys | 100 | 100 |
| PVP | 100 | 100 |
| PEG | 100 | 100 |
| PVA | 100 | 100 |
| Dex | 100 | 100 |
| PAA | 100 | 100 |
| BSA | 100 | 100 |
| Gelatin | 100 | 100 |
| (−) | 100 | 100 |

## References


(1) Chan, W. C.; White, P. D. *Fmoc Solid Phase Peptide Synthesis*; Oxford University Press, 2000.

(2) Suzuki, S.; Sawada, T.; Serizawa, T. Identification of Water-Soluble Polymers through Discrimination of Multiple Optical Signals from a Single Peptide Sensor. *ACS Appl. Mater. Interfaces* **2021**, *13* (47), 55978–55987.
